# Functional Insights into Recombinant TROSPA Protein from *Ixodes ricinus*

**DOI:** 10.1371/journal.pone.0076848

**Published:** 2013-10-18

**Authors:** Marek Figlerowicz, Anna Urbanowicz, Dominik Lewandowski, Jadwiga Jodynis-Liebert, Czeslaw Sadowski

**Affiliations:** 1 Institute of Bioorganic Chemistry, Polish Academy of Sciences, Poznan, Poland; 2 Department of Toxicology, Poznan University of Medical Sciences, Poznan, Poland; 3 Institute of Computing Science, Poznan University of Technology, Poznan, Poland; University of Kentucky College of Medicine, United States of America

## Abstract

Lyme disease (also called borreliosis) is a prevalent chronic disease transmitted by ticks and caused by *Borrelia burgdorferi s. l.* spirochete. At least one tick protein, namely TROSPA from *I. scapularis*, commonly occurring in the USA, was shown to be required for colonization of the vector by bacteria. Located in the tick gut, TROSPA interacts with the spirochete outer surface protein A (OspA) and initiates the tick colonization. *Ixodes ricinus* is a primary vector involved in *B. burgdorferi s. l.* transmission in most European countries. In this study, we characterized the capacities of recombinant TROSPA protein from *I. ricinus* to interact with OspA from different *Borrelia* species and to induce an immune response in animals. We also showed that the N-terminal part of TROSPA (a putative transmembrane domain) is not involved in the interaction with OspA and that reduction of the total negative charge on the TROSPA protein impaired TROSPA-OspA binding. In general, the data presented in this paper indicate that recombinant TROSPA protein retains the capacity to form a complex with OspA and induces a significant level of IgG in orally immunized rats. Thus, *I. ricinus* TROSPA may be considered a good candidate component for an animal vaccine against *Borrelia*.

## Introduction

Hard ticks of the genus *Ixodes* are ectoparasites of vertebrates. Ticks feed on animal blood, and consequently, they may function as vectors for numerous pathogenic microorganisms of vertebrates. Among other microbes, ticks transmit *Borrelia burgdorferi sensu lato* (*s. l.*), the causative agent of Lyme disease, also called borreliosis. *B. burgdorferi s. l.* is a phylogenetic group clustering numerous *Borrelia* species including the most common *B. burgdorferi sensu stricto (s. s.)*, *B. afzelii* and *B. garinii* [1]. These spirochetes are widely spread all over the temperate zone of the northern hemisphere [2], [3], [4], [5], [6], [7]. *Ixodes ricinus* is a primary vector for *B. burgdorferi s. l.* in most European countries, while *Ixodes scapularis* spreads mainly *B. burgdorferi s. s.* - a bacterial species typical for endemic areas of the USA [1]. The percentage of *Borrelia*-infected *I. ricinus* ticks in Europe ranges from a few to several dozen, depending on the region [4], [7], [8]. In the USA, the prevalence of *Borrelia* in ticks reaches up to sixty percent [3], [9], [10]. Borreliosis in humans is a serious chronic disease affecting multiple organ systems including the nervous system, cardiovascular system, muscles and joints. However, humans are only accidental hosts for *Borrelia* spirochetes. In a natural environment, the reservoir of *Borrelia* are small, wild vertebrates, mainly rodents [1].

*Borrelia* from the blood of an infected animal enters the tick during feeding. At least one tick protein, namely TROSPA from *I. scapularis*, was proven to be crucial for the colonization of the vector by bacteria. Located in the tick gut, TROSPA interacts with the spirochete outer surface protein A (OspA) [11]. The interaction between these two proteins allows the spirochetes to associate with the gut tissue, which is the first step of tick colonization. The data suggest that TROSPA is subjected to extensive post-translational modifications *in vivo*. Most likely glycosylated, 55 kDa TROSPA protein was found in *I. scapularis*. A 16 kDa protein was formed when the TROSPA encoding gene from *I. scapularis* was expressed in a bacterial system. Although this protein lacked post-translational modifications, it was capable of binding to OspA in a similar manner as to native glycosylated TROSPA [11]. Recently, the TROSPA homolog from *I. persulcatus* has also been identified [12]. This protein was predicted to possess a putative transmembrane helix at the N-terminus [11], [12]. Although the interaction between *Ixodes* TROSPA and spirochete OspA is indispensable for the circulation of *Borrelia* between the vector and the host, little is known about the nature of this interaction. In addition, the majority of the data collected so far have been obtained using TROSPA from *I. scapularis*, which is typical for the USA but not for Europe.

To date, approaches to prevent *Borrelia* infections have been focused on bacterial outer surface proteins [13], [14], [15]. For example, the vaccinations with OspA conferred a high level of resistance to borreliosis in the USA [13], [14]. Unfortunately, some disadvantages of the outer surface proteins-based vaccine were reported. These included high variability of antigens in the individual *Borrelia* strains (causing OspA-based vaccine to confer the immunity only to one particular strain of *Borrelia*), antigenic differences among spirochetes occurring in different parts of the world (especially in Europe) and adverse side effects (e.g., arthritis) [1], [13], [15]. As a result, an idea was proposed to develop a tick protein-based vaccine against borreliosis. In addition, the possibility of a *Borrelia* reservoir vaccination has been considered [15]. The involvement of *Ixodes* TROSPA in tick colonization suggests this protein as a promising component of a vaccine to prevent the transmission of *B. burgdorferi*. The anti-TROSPA antibodies present in animal blood would compete with *Borrelia* for TROSPA binding in the tick gut and consequently by impeding tick gut colonization by spirochetes restrict their prevalence. The use of TROSPA in a vaccine is strongly supported by reports showing that the colonization of ticks by spirochetes was significantly impaired when the parasites fed on *Borrelia*-infected mice that had been injected with TROSPA antisera. Additionally, the repression of TROSPA gene expression by RNA interference significantly reduced *B. burgdorferi* adherence to the gut of *I. scapularis* [11].

The evidence presented above revealed the importance of the TROSPA protein to the *Borrelia* life cycle and showed that one can limit *Borrelia* transmission by inhibiting TROSPA-OspA binding. Accordingly, our studies were focused on exploring the details of the interaction between these proteins. Earlier this issue was investigated using TROSPA from *I. scapularis* and OspA from *B. burgdorferi s. s*., both typical for North America. Considering the fact that bacterial outer surface proteins may significantly differ depend on *Borrelia* geographical localization, we decided to use in our experiments TROSPA from *I. ricinus*, the prevalent vector of *B. burgdorferi s. l.* in Europe, and OspA from three bacterial species also typical for Europe: *B. garinii*, *B. afzelii* and *B. burgdorferi s. s*. We cloned TROSPA gene from *I. ricinus* and three OspA genes from above mentioned *Borrelia* species and elaborated on the methods of the production of these proteins in a bacterial system. We showed that the recombinant TROSPA was able to form complexes with its bacterial partners, three OspA proteins. Interestingly, we observed that OspA proteins from different bacterial species showed various capacities to bind TROSPA. To find out which part of TROSPA is involved in interaction with OspA and what is the nature of this interaction, we generated a series of TROSPA mutants and assessed their ability to bind OspA. Finally, we determined the immunogenic properties of recombinant TROSPA by using it to induce an immune response in rats.

## Results

### TROSPA Expression in Bacterial and Plant Systems

**Figure 1:**
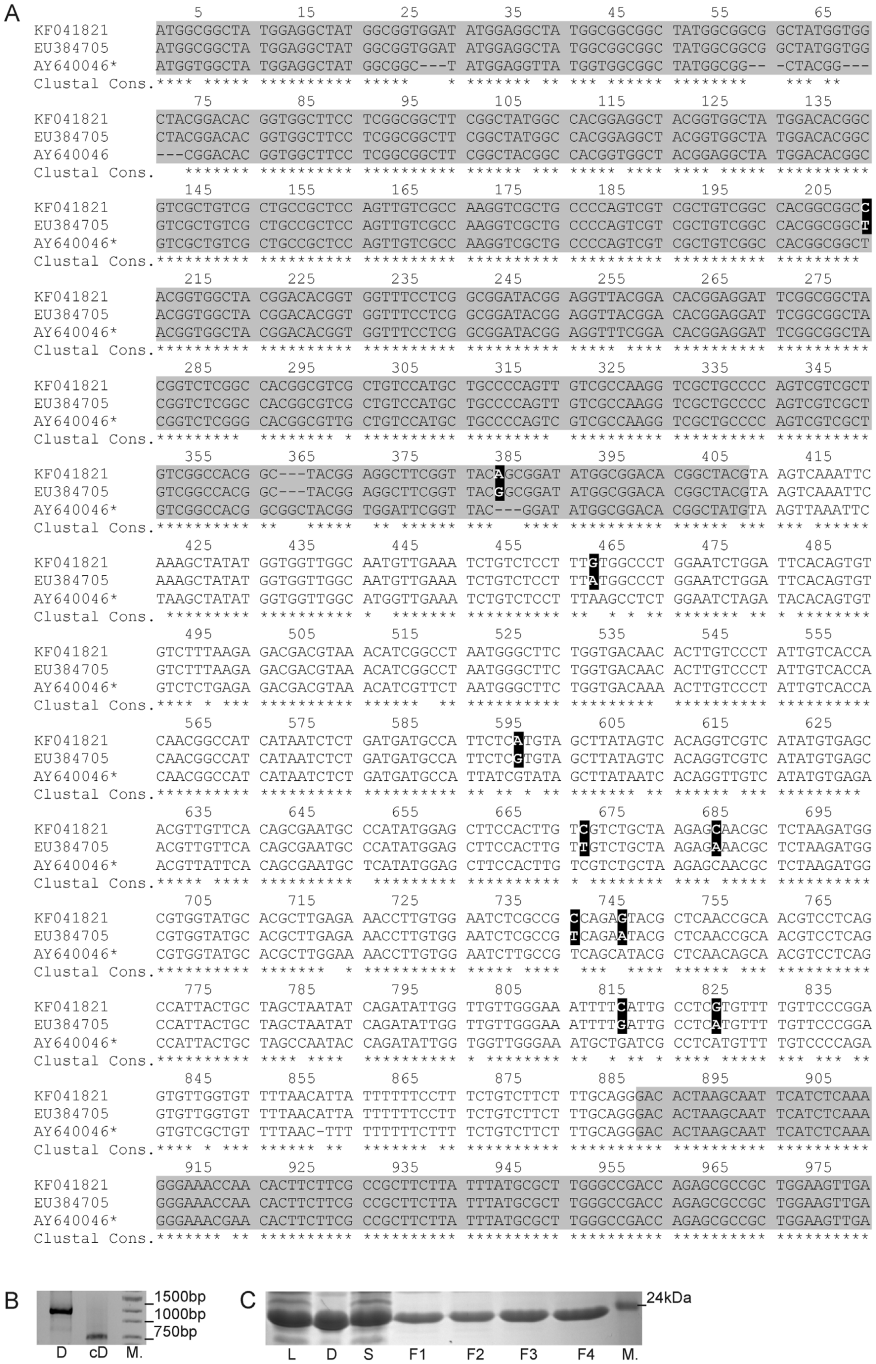
*I. ricinus* TROSPA coding sequence and the product of its expression in *E. coli.* **A**- Comparison of the DNA sequence encoding *I. ricinus* TROSPA KF041821 (Polish strain), reference sequence GB number EU384705 and a part of *I. scapularis* TROSPA gene GB number AY640046 (AY640046* - coding regions with intron are presented). The coding regions are marked in grey, the identical nucleotides are indicated with asterisks, different nucleotides are indicated with black boxes and white letters (with the respect to *I. ricinus* sequences). **B** - Image of PCR-amplified *I. ricinus* TROSPA DNA (D) and cDNA (cD) resolved in an agarose gel. M - DNA weight marker. **C** - Image of recombinant *I. ricinus* TROSPA protein preparations resolved by SDS-PAGE. L - *E. coli* lysate, **D** - cell debris after centrifugation of the lysate, S - supernatant after centrifugation of the lysate, F1–F4 - fractions washed out from the Ni-NTA column, M - weight marker.

In the first stage of our studies, we attempted to produce TROSPA protein in bacterial and plant cells. To this end, the fragment of the TROSPA gene lacking the 3′ and 5′ noncoding sequences was amplified via PCR using *I. ricinus* genomic DNA and primers that were designed based on the reference sequence (EU384705). The PCR product was 977 nt long, as expected. We have already submitted this sequence to GenBank (accession number KF041821). It encompassed two exons and a centrally located intron and showed 98.97% identity (ClustalW) with the reference sequence. The reference and the amplified fragment of the TROSPA gene differed by 10 nucleotides (Fig. 1A). Eight of these were located in the intron and two were within the 5′ exon, but the resulting amino acid sequence remained unchanged.

The predicted amino acid sequence of TROSPA contained a few potential posttranslational modification signals, including O- and C-glycosylation [11], [12]. To obtain posttranslationally modified and unmodified forms of the protein, constructs for the production of TROSPA in plants and in bacteria were prepared. First, the amplified fragment of the TROSPA gene was cloned into a binary vector (pGreenII) under the control of a 35S promoter. The obtained plasmid pGT was used for the transient expression of TROSPA in *N. benthamiana*. Five days after agroinfiltration, total RNA was extracted from the plants, and the TROSPA cDNA was amplified by RT-PCR. Sequencing of the cDNA confirmed the correct splicing of the TROSPA sequence in *N. benthamiana* (Fig. 1B). The cDNA was then cloned into a pET200 expression vector, which was used for TROSPA production in a bacterial system (Fig. 1C). The expected molecular mass of the recombinant TROSPA protein (calculated based on its amino acid composition) is about 16 kDa. A similar molecular mass has been earlier calculated for recombinant TROSPA from *I. scapularis* [11]. The MALDI-TOF MS analysis (data not shown) demonstrated that the molecular mass of recombinant TROSPA from *I. ricinus* is 16,1kDa. Thus the obtained result was consistent with the above calculation. Interestingly, we found that recombinant TROSPA migrated together with 20–25 kDa proteins when separated by SDS-PAGE (Fig. 1C). Similar phenomenon was also reported for recombinant TROSPA from *I. scapularis* [11].

Recombinant TROSPA, after purification on a Ni-NTA column and removal of the N-terminal tag, was used to generate TROSPA antisera. The sera were used for the detection of TROSPA in the total protein extract from agroinfiltrated fragments of *N. benthamiana* leaves. Protein was isolated from plants at 6, 8, and 10 days after agroinfiltration [16]and subjected to western blot analysis. Surprisingly, TROSPA protein was not detected in *N. benthamiana* leaves (data not shown).

### Interaction of TROSPA from *I. ricinus* with OspA from *Borrelia burgdorferi*

**Figure 2:**
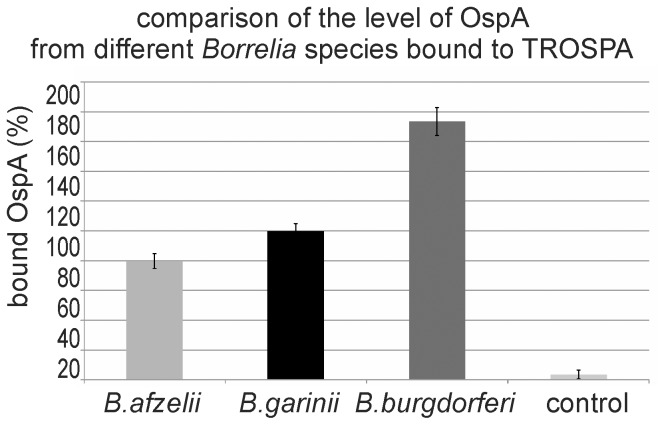
Comparison of the relative level of OspA protein from different *Borrelia* species bound to TROSPA. TROSPA protein-coated ELISA microplates were incubated with OspA protein (c = 30 µg/ml) from *B. afzelii, B. garinii* or *B. burgdorferi s. s.* Bound OspA was subsequently detected using rabbit *Borrelia*-specific polyclonal IgG (primary antibody) and AP-conjugated anti-rabbit polyclonal IgG (secondary antibody). Absorbance of the soluble product of the alkaline phosphatase reaction was measured with reference to blank samples containing no OspA. As a control, analogous assays with OspC from *B. garinii* were performed. The level of individual OspA protein binding was always determined based on 16 reactions (for details see Materials and methods). For each protein pair arithmetic mean of absorbance and SD was calculated and expressed in percentage, assuming that the level of TROSPA binding with OspA from *B. garinii* is 100%. The differences between bound OspA from *B. garinii* and OspA from *B. afzelii* (p = 0,0003) or OspA from *B. garinii* and OspA from *B. burgdorferi s. s.* (p<0,0001) were statistically significant.

As mentioned above, the *I. scapularis* TROSPA protein is required for tick colonization. During this process, TROSPA, located in the tick gut, specifically binds the OspA protein anchored in the *B. burgdorferi* outer membrane [11]. Accordingly, we predict that the TROSPA homolog from *I. ricinus* is also capable of binding to OspA. To verify this hypothesis, we assessed the interactions between recombinant TROSPA from *I. ricinus* and OspA from *B. burgdorferi sensu stricto*, *B. garinii* or *B. afzelii*. Serial dilutions of OspA preparations were added to TROSPA-coated ELISA microplates. The most significant differences between the bindings of the three OspAs to TROSPA were observed at OspA concentrations of 30 µg/ml (data not shown). The level of OspA binding was determined using rabbit *Borrelia*-specific polyclonal IgG (primary antibody) and AP-conjugated anti-rabbit polyclonal IgG (secondary antibody). Western blot analysis previously demonstrated that the primary antibody has equal capacity to bind with all three types of OspA (data not shown). Absorbance of the soluble product of the AP-catalyzed reaction was measured with reference to the blank samples that contained no OspA. The results of these assays showed a concentration-dependent formation of the OspA-TROSPA complex. Moreover, we observed that the OspA proteins derived from various *Borrelia* species have different abilities to bind *I. ricinus* TROSPA (p≤0,0003) (Fig. 2). As a control, analogous assays with OspC from *B. garinii* were performed. These assays demonstrated no specific OspC-TROSPA binding.

### Search for TROSPA Motifs Critical for TROSPA-OspA Interaction

**Figure 3:**
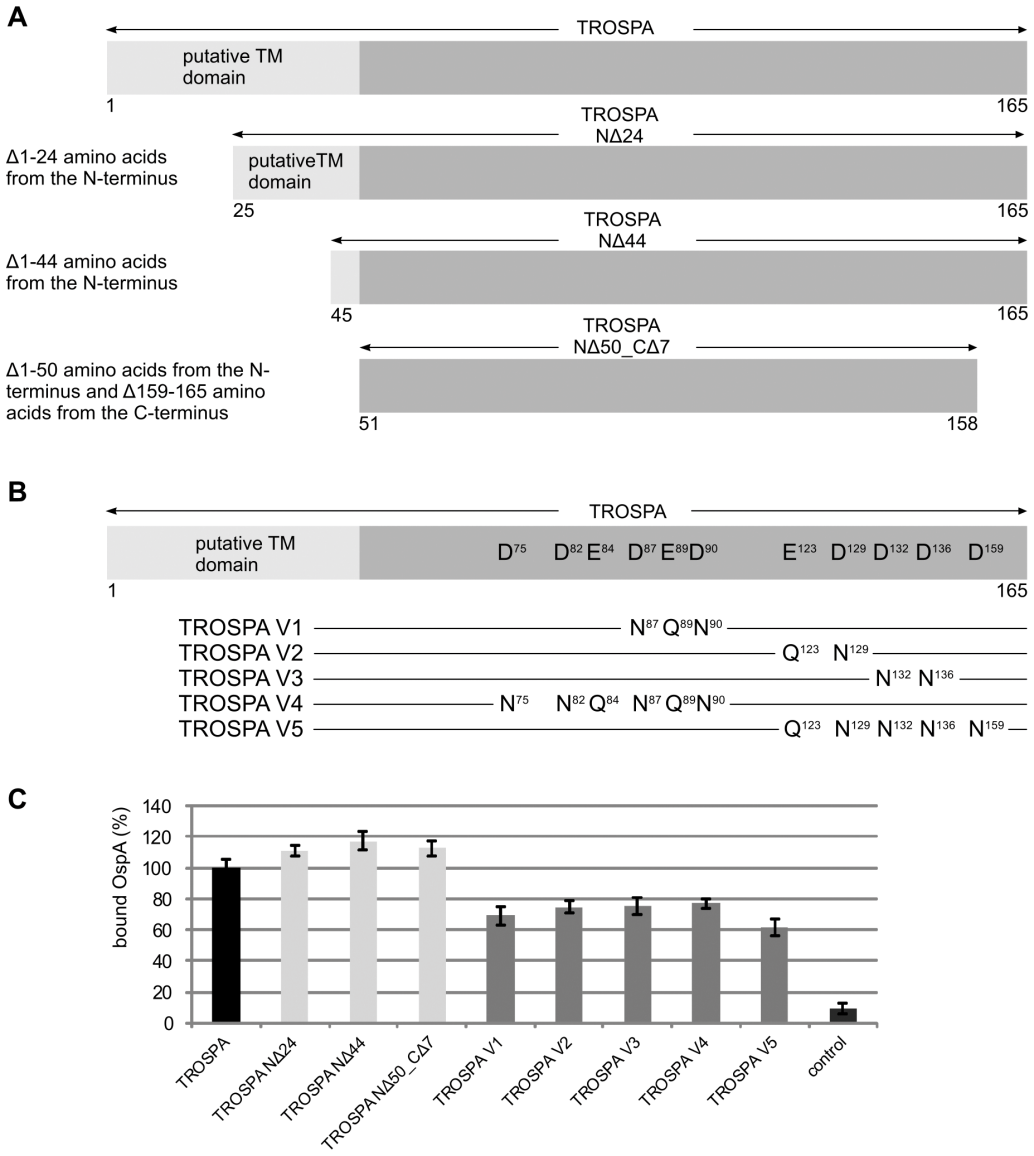
Schematic depiction of the two series of *I. ricinus* TROSPA mutants and the level of bound OspA protein from *Borrelia garinii*. **A**- TROSPA deletion mutants. In the NΔ24 mutant, amino acids 1 to 24 were deleted; in the NΔ44 mutant, amino acids 1 to 44 were deleted; in the NΔ50_CΔ7 mutant, amino acids 1 to 50 and 159 to 165 were deleted. **B** - TROSPA substitution mutants. Selected negatively charged amino acid residues were changed to neutrally charged amino acids: in the V1 TROSPA mutant, aspartic acids 87 and 90 were replaced with asparagine, and glutamic acid 89 was replaced with glutamine; in the V2 TROSPA mutant, glutamic acid 123 was replaced with glutamine, and aspartic acid 129 was replaced with asparagine; in the V3 TROSPA mutant, aspartic acids 132 and 136 were replaced with asparagines; in the V4 TROSPA mutant, aspartic acids 75, 82, 87 and 90 were replaced with asparagines, and glutamic acids 84 and 89 were replaced with glutamines; in the V5 TROSPA mutant, glutamic acid 123 was replaced with glutamine, and aspartic acids 129, 132, 136 and 159 were replaced with asparagines. **C** - Comparison of the relative level of OspA from *B. garinii* bound to the TROSPA mutants. TROSPA- or TROSPA mutants- coated ELISA microplates were incubated with *B. garinii* OspA protein (C = 30 µg/ml, 100 µl/well). Bound OspA was subsequently detected using rabbit *Borrelia*-specific polyclonal IgG (primary antibody) and AP-conjugated anti-rabbit polyclonal IgG (secondary antibody). Absorbance of the soluble product of the alkaline phosphatase reaction was measured with reference to blank samples containing no OspA. For each TROSPA or TROSPA mutant the level of OspA binding was determined based on 16 reactions (for details see Materials and methods). For each protein pair arithmetic mean of absorbance and SD was calculated and expressed in percentage, assuming that the level of TROSPA binding with OspA from *B. garinii* is 100%. The differences of OspA binding among unmutated TROSPA and the deletion mutants were statistically significant (p = 0,0002 for the NΔ24 mutant, p<0,0001 for the NΔ44 mutant and p = 0,0003 for the NΔ50_CΔ7 mutant). All the mutants bearing the reduced total negative charge (the V1–V5 mutants) displayed significantly lower affinity for OspA (p<0,0001) compared to control unmutated TROSPA.

A bioinformatics analysis of the TROSPA amino acid sequence indicated that an N-terminal transmembrane domain is present in proteins from *I. scapularis* and *I. persulcatus* [11], [12]. Thus, it is not likely that this domain is involved in the interaction with OspA. In addition, there are indications that TROSPA-OspA interactions are electrostatic in nature; the overall charge of TROSPA protein is −12 and for OspA is +5 (in PBS, pH 7,4). To verify if these observations also apply to *I. ricinus* TROSPA and to identify which amino acid residues are important for interaction with OspA, two series of mutants were created. The first series included two N-terminal deletion mutants: TROSPA_NΔ24 mutant lacking amino acid 1–24 and TROSPA_NΔ44 lacking amino acids 1–44. A third mutant was devoid of amino acids from both the N- and C-termini: TROSPA_NΔ50_CΔ7 lacking amino acids 1–50 and 159–165 (Fig. 3A). The 7 amino acid long C-terminal fragment of TROSPA_NΔ50_CΔ7 was removed due to its tendency to spontaneously degrade during the purification procedure. The degradation process was confirmed by MALDI-TOF MS analysis. The second series (TROSPA V1–V5) included mutants with substitutions distributed in different regions of the TROSPA protein. In these mutants, amino acid residues bearing negative charge were replaced with their neutral analogs. In the TROSPA V1 mutant, D87 and D90 were replaced with N, and E89 was replaced with Q; in the TROSPA V2 mutant, E123 was replaced with Q, and D129 was replaced with N; in the TROSPA V3 mutant, D132 and D136 were replaced with N; in the TROSPA V4 mutant, D75, D82, D87 and D90 were replaced with N, and E84 and E89 were replaced with Q; in the TROSPA V5 mutant, E123 was replaced with Q, and D129, D132, D136 and D159 were replaced with N (Fig. 3B).

All of these mutants were expressed in a bacterial system and purified as previously described for wild type protein. The influence of the mutations on the ability of TROSPA to bind OspA was determined by immunoassay. Microplates were coated with TROSPA mutants (C = 5 µg/ml, 100 µl/well) and probed with *B. garinii* OspA at a concentration of approximately 30 µg/ml (100 µl/well). *B. garinii* OspA was chosen because among the three previously tested OspA proteins, it showed a moderate level of affinity for TROSPA. The results (Fig. 3) revealed that deletion of the N-terminal 24 or 44 amino acids of TROSPA, or deletion of the whole predicted transmembrane domain (50 N-terminal amino acids) together with 7 amino acids from the C-terminus, slightly increased the capacity of TROSPA to bind OspA (by 11%, 17% and 13%, respectively, compared to wt TROSPA; p≤0,0003) (Fig. 3C). In contrast, the mutants bearing the reduced total negative charge displayed significantly lower affinity for OspA (by 31%, 25%, 24%, 23% and 39% for V1–V5 mutants, respectively; p<0,0001) compared to control wt TROSPA. However, none of the V1–V5 series of mutants showed a complete loss of binding affinity for OspA.

### *I. ricinus* TROSPA as a Candidate for a Vaccine Against Borreliosis

TROSPA protein has been considered a potential antigen for a vaccine against borreliosis [11], [12], [17]. It was shown that in animals experimentally infected with *Borrelia*, administration of *I. scapularis* TROSPA antisera reduced (up to 75%) the colonization of the tick and subsequently the number of spirochetes that were transmitted to the next mammalian host [11].

**Figure 4:**
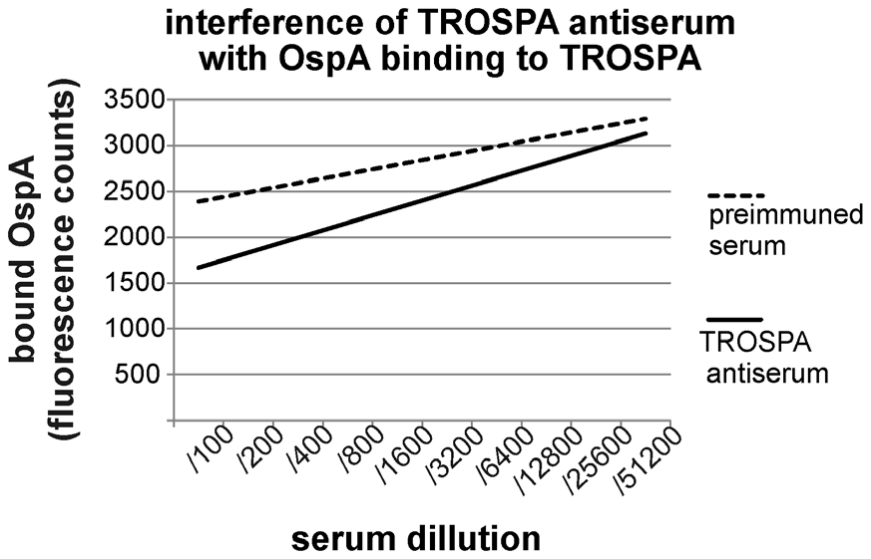
Interference of the TROSPA antiserum with the level of OspA bound to TROSPA. *I. ricinus* TROSPA protein-coated ELISA microplates were incubated with the serial dilutions of rabbit TROSPA antiserum or rabbit preimmune antiserum. After washing, the plates were treated with *B. garinii* OspA protein (C = 30 µg/ml, 100 µl/well), which was subsequently detected by FITC-conjugated anti*-Borrelia* goat polyclonal IgG. The X axis represents serial sera dilutions, and the Y axis represents the level of fluorescence counts. The level of TROSPA-OspA binding was determined for ten rabbit TROSPA antiserum or preimmune serum concentrations. The level of OspA binding was determined as the arithmetic mean of fluorescence counts based on 16 reactions performed for every TROSPA antiserum or preimmune serum dilution (for details see Materials and methods). The reduction of fluorescence counts was statistically significant (p<0,01) for all serum dilutions except the highest one.

In our studies, TROSPA antiserum, generated using recombinant *I. ricinus* TROSPA, was used to examine the influence of anti-TROSPA immunoglobulin on the formation of the TROSPA-OspA complex. The experiments were conducted similarly for the TROSPA mutants. However, before OspA was added to the TROSPA-coated microplates, the plates were treated with serial dilutions of *I. ricinus* TROSPA antiserum (Fig. 4). In a control experiment, TROSPA-coated microplates were treated with preimmune rabbit serum. After plates were incubated with *B. garinii* OspA protein, the level of OspA binding with TROSPA was determined using FITC-conjugated anti-*Borrelia* IgG and fluorescence counting. TROSPA-coated plates treated only with FITC-conjugated anti*-Borrelia* IgG were used as reference samples. As expected, significantly less OspA was bound to the TROSPA-coated microplates treated with TROSPA antiserum compared to microplates treated with control preimmune serum. A 30% reduction of TROSPA-OspA binding was observed when TROSPA antiserum was applied at the highest concentration (p = 0,001).

**Figure 5:**
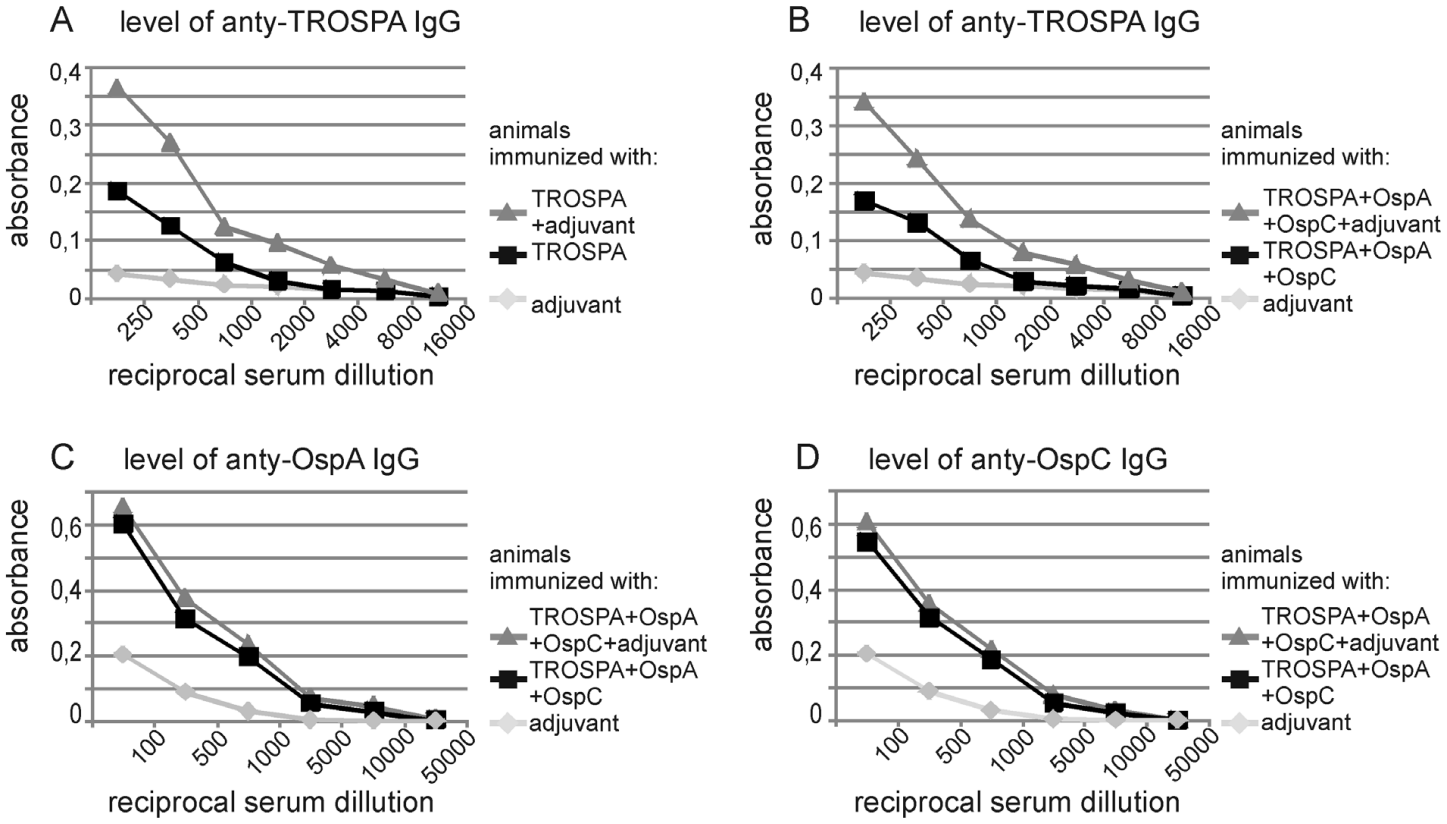
Titers of IgG detected by ELISA in the sera of orally immunized rats. Rats were immunized on days 0, 14, and 28; sera were collected on day 42. Next, sera were serially diluted and subjected to ELISA tests. **A** - The level of specific IgG in groups of 5 rats immunized with TROSPA or TROSPA with adjuvant. **B–D** - The level of specific IgG in groups of 5 rats immunized with TROSPA, OspA and OspC without or with adjuvant. The control group of 5 rats was immunized with adjuvant only. The X axis represents the reciprocal sera dilutions, and the Y axis represents the level of absorbance of the color product of the alkaline phosphatase reaction. The levels of IgG were calculated as the arithmetic means of absorbance measured in ELISA tests performed for the serially diluted sera from each group of 5 animals. The levels of IgG were determined based on 15 reactions performed for every serum dilution (for details see Materials and methods).

To further examine the antigenic properties of recombinant TROSPA protein from *I. ricinus*, we used TROSPA for oral immunization of animals. Two types of immunizations were performed, with the use of the TROSPA protein alone or with the use of the TROSPA protein together with *B. garinii* OspA and OspC proteins. A combined vaccine composed of these three proteins would generate the antibodies working against *Borrelia* infection on different stages of its life cycle. We wanted to find out if the application of three different antigens together would influence the level of the generated anti-TROSPA antibodies. The first group of 10 rats was immunized with TROSPA protein (including 5 individuals immunized with the addition of an adjuvant). The second group of 10 rats was immunized with TROSPA protein in combination with *B. garinii* OspA and OspC proteins, including 5 individuals immunized with the addition of an adjuvant. The experiments with or without adjuvant were performed to address the question to what extent the immunogenic properties of TROSPA would be enhanced by adjuvant addition. The control group of 5 rats was immunized with the adjuvant alone. Rats were immunized three times, on days 0, 14, and 28. During the experiment, the rats were observed and weighed every seven days to exclude negative effects of immunization on their health. After 42 days, serum sampling was performed. Sera were serially diluted and an ELISA was used to assess the level of induced IgG (Fig. 5). In both groups of animals, high levels of anti-TROSPA IgG were detected, although the levels of anti-OspA and anti-OspC IgG were significantly higher (p = 0,001). The level of anti-TROSPA IgG was defined as the biggest reciprocal serum dilution which showed higher absorbance readings than the control serum in the ELISA [18]. In TROSPA-immunized or TROSPA-, OspA- and OspC-immunized animals, the level of anti-TROSPA IgG reached 2500 (without adjuvant) or 5000 (with adjuvant). The levels of anti-OspA and anti-OspC IgG (also defined as the biggest reciprocal serum dilution showing higher absorbance than the control) were as high as 10000.

## Discussion

To initiate circulation in the natural environment, *B. burgdorferi* colonizes ticks [11]. One of the key factors involved in this process is a tick gut protein, TROSPA. TROSPA retains bacteria in the vector digestive tract by binding the spirochete surface protein *I. ricinus* is the widest spread vector of *Borrelia* in Europe [1], [4], [7], [8], [19]. Accordingly, the main object of our studies was the TROSPA protein from this species. First, we amplified and cloned a fragment of the TROSPA gene. This fragment contained two exons encoding whole TROSPA and one intron; the cloned fragment was nearly identical (98.97%) with the reference sequence EU384705. The amino acid sequences deduced from both amplified and reference DNA sequences were identical (Fig. 1A). Next, we attempted to produce TROSPA protein in plant and bacterial expression systems. We found that TROSPA was efficiently produced in the *E. coli* cells, while it was not detected in the total protein isolated from the plant cells. However, the presence of correctly spliced mRNA was confirmed (Fig. 1B). TROSPA was detected on western blots using sera generated in animals injected with recombinant protein that was produced in bacteria. Earlier studies of TROSPA isolated from *I. scapularis* suggest that this protein is extensively glycosylated [11], [12]. Accordingly, it could be that TROSPA was undetected in plants because the antibodies were targeted against non-glycosylated protein. This is most likely not the case because the above mentioned study showed that antisera raised against recombinant TROSPA (from *I. scapuaris*) produced in *E. coli* specifically reacted with the glycosylated native TROSPA protein from *I. scapularis* gut cell lysates [11]. Thus, we reason that native TROSPA from *I. ricinus* should also be detected with antisera raised against recombinant protein. Accordingly, we propose that TROSPA was not effectively produced in our plant system or that it was produced and then rapidly degraded by plant proteases. Similar phenomena have been reported for many other bacterial or animal proteins that were expressed in various plant systems [20].

We showed that highly purified, non-glycosylated recombinant *I. ricinus* TROSPA can be obtained using affinity chromatography. Next, we examined if this protein retains its natural capacity to bind bacterial OspA. The most abundant *Borrelia* species in Europe are: *B. burgdorferi sensu stricto*, *B. garinii* and *B. afzelii* [1], thus, in our experiments we used recombinant OspA proteins derived from these species. We observed that the TROSPA-OspA complex was formed in all cases; however, the amount of complex was slightly different for the three proteins (Fig. 2). We conclude that *I. ricinus* TROSPA can be involved in tick colonization by at least these three species of *Borrelia*, however the efficacy of colonization may varies depending on *Borrelia* species. Similar observations have been made by other researchers. For example, Pal and coworkers reported that OspA from *B. burgdorferi s. s.* strains N40 and 25015 were both able to bind to the tick gut extract, but the 25015 OspA formed a more stable complex [21]. If there is such a variation within one bacterial species, the differences observed for OspA derived from different species are not surprising. The question is whether these differences result only from OspA-TROSPA binding or also from interactions between OspA-OspA. It was demonstrated that OspA proteins are capable of forming homopolymeric complexes [21]. In this way, the number of the OspA proteins, and consequently bacterial cells, that are attached to the tick gut epithelial cells can be increased. Taking into account the conservation of TROSPA and the variability of OspA, one can hypothesize that the efficiency of vector colonization by *Borrelia* to a higher extent depends on the properties of bacterial than tick encoded protein. In other words, it is possible that the type of OspA may influence the local prevalence of *Borrelia* species.

So far, the molecular determinants of TROSPA-OspA recognition remain unknown. To investigate the nature of the TROSPA-OspA interaction, we prepared several TROSPA mutants. Bioinformatics analysis of the TROSPA protein amino acid sequence indicated the presence of an N-terminal transmembrane domain and an overall negative charge of the protein (−12). Similar analysis of OspA revealed that it carries a positive charge (+5). Therefore, we hypothesized that electrostatic interactions may play a significant role in the TROSPA-OspA binding. To verify this hypothesis, we designed two series of TROSPA mutants. The first series included two mutants with an N-terminal deletion encompassing a large fragment or the whole putative transmembrane domain (Fig. 3A). The second series included mutants in which selected amino acid residues bearing negative charge were replaced with their neutral analogs (Fig. 3B). The analysis of the interaction between the TROSPA deletion mutants and OspA protein from *B. garinii* confirmed that the N-terminal part of TROSPA does not play a role in OspA binding (Fig. 3C). The removal of this domain, in whole or part, did not significantly decrease the capacity of TROSPA to bind OspA. In contrast, analysis of the capacities of TROSPA V1–5 mutants to bind with OspA was not entirely conclusive. All the substitution mutants showed significantly lower affinity for OspA compared to the wild type TROSPA (Fig. 3C). However, none of the mutations caused TROSPA to be incapable of binding with OspA. Similarly, amino acid substitutions introduced by Pal and coworkers to OspA regions that strongly bound to tick gut extract did not definitively identify the character of the interaction [21]. Thus, the nature of the TROSPA-OspA interaction seems to be more complex than electrostatic interactions alone. Because the native TROSPA protein can be post-translationally modified, one cannot exclude that these modifications also participate in these interactions. To examine this possibility, the stability of the complexes formed by native and recombinant TROSPA with OspA should be compared.

In Europe, OspA shows a significant level of diversity [1], [19], [22], this being one of the reasons underlying the ineffective diagnosis and prevention of Lyme disease. In contrast to OspA, *I. ricinus* TROSPA seems to be a very conservative protein, taking into account GenBank data (Fig. S1). In addition, TROSPA clearly binds with OspA from different strains of *Borrelia*, a crucial step in the spirochete life cycle. These features of TROSPA make the *I. ricinus* protein a good candidate for a vaccine against *Borrelia*. We propose that a vaccine containing this protein can be used for the immunization of wild animals, which are the natural reservoir for the bacteria. This would serve to lower the infection rates of *Ixodes* ticks and consequently reduce the risk of *Borrelia* transmission to humans. Our studies also showed that oral immunization of animals with recombinant *I. ricinus* TROSPA protein induced a high level of IgG (Fig. 5), suggesting strong immunogenic properties of this protein [23]. We further demonstrated that antibodies against TROSPA can outcompete OspA protein in binding with TROSPA (Fig. 4).

In conclusion, the data collected here indicate that recombinant TROSPA protein from *I. ricinus* produced in a bacterial system retains the ability to interact with OspA from three most abundant European species of *Borrelia*. The different affinity of TROSPA to OspA derived from various bacterial species suggests that this interaction may influence the occurrence of particular *Borrelia* species in a given geographic area. The N-terminal part of TROSPA is not involved in the interaction with OspA, which is consistent with *in silico* predictions. However, further studies are required to precisely determine the amino acids or structural motifs that are involved in forming TROSPA - OspA complex, because it is only partially determined by the electrostatic interactions. Finally, *I. ricinus* TROSPA protein exhibits a number of features making it a good candidate for a vaccine for the wild life in order to decrease the natural reservoir of *Borrelia*. These features are: conservative amino acid sequence, ability to induce a high level of antibodies in orally immunized animals and ability to compete with OspA for TROSPA binding.

## Materials and Methods

### Ethics Statement

The experiments involving animals were performed in strict accordance with standards of European Union legislation. The protocols were evaluated and approved by the Local Ethical Committee for Animal Experimentation in Poznan, Poland (Permit Number: 52/2008). The rats were anaesthetized by intramuscular injection of ketamine and xylazine (30–35 and 40–90 mg/kg, respectively), all efforts were made to minimize animal suffering and to reduce the number of animals used.

### Construction of Plasmids for the Production of TROSPA, OspA and OspC Proteins in a Bacterial System

*I. ricinus* ticks were obtained from Dr. Beata Wodecka from the Department of Genetics, Szczecin University, Szczecin, Poland. Genomic DNA of *I. ricinus* was extracted from the ticks using a QIAGEN DNeasy Blood & Tissue Kit. A fragment of the TROSPA gene located between the start and stop codons (a fragment containing two exons and a centrally positioned intron) was amplified by PCR using primers T5′ and T3′. The primers were designed based on the sequence of the *I. ricinus* TROSPA gene (GenBank sequence number EU034646.1). The sequence of the 977-nt long PCR product was submitted to GenBank, accession number KF041821. The PCR product was ligated into the pGreenII vector using *Eco*RI and *Sma*I restriction sites. The pGreenII-based construct bearing the fragment of the TROSPA gene under the 35S-CaMV promoter (named pGT) was used to transform the *Agrobacterium tumefaciens* GV3101 strain. The resultant *A. tumefaciens* GV3101-pGT strain was applied to 6-week old *Nicotiana benthamiana* plants for agroinfiltration. Briefly, after overnight culture, 1 ml of *A. tumefaciens* GV3101-GT was centrifuged for 3 min at 8 krpm at room temperature, and the pellet was dissolved in 10 ml of infiltration buffer (10 m*M* MES-NaOH pH 5.5, 10 m*M* MgSO_4_). The undersides of *N. benthamiana* leaves were infiltrated with syringe. Agroinfiltrated plants were maintained at 23°C in a growth chamber with a 16-h photoperiod. Five days post agroinfiltration, total RNA was extracted from *N. benthamiana* using a QIAGEN RNeasy Plant Mini Kit. TROSPA cDNA was amplified by reverse transcription and PCR using TF and T3′ primers and cloned into a pET vector using a Champion™ pET200 Directional TOPO Expression Kit (Invitrogen) according to the manufacturer’s instructions.

DNA encoding either *B. burgdorferi sensu sticto* (ZS7 strain), *B. afzelii* or *B. garinii* OspA (Materials S1B) and *B. garinii* OspC (GB accession number: D49498.1) were provided by Dr. Beata Wodecka (Department of Genetics, Szczecin University, Szczecin, Poland). The OspA genes were amplified via PCR using primers AF and AR, and the OspC gene was amplified using primers CF and CR. The PCR products were cloned into a pET expression vector using a Champion™ pET200 Directional TOPO® Expression Kit according to the manufacturer’s instructions. All the primer sequences are in Materials S1A.

### TROSPA Mutant Preparation

To obtain plasmids encoding TROSPA deletion mutants (TROSPA M1_A24del designated as TROSPA_NΔ24, TROSPA M1_M44del designated as TROSPA_NΔ44, TROSPA M1_S50del_D159_S165del designated as TROSPA_NΔ50_CΔ7), the selected fragments of TROSPA cDNA were obtained by PCR using the following primers: T24F and TR to amplify DNA encoding TROSPA shortened by 24 amino acids from the N-terminus; T44F and TR to amplify DNA encoding TROSPA shortened by 44 amino acids from the N-terminus; T50F and T7R to amplify DNA encoding TROSPA shortened by 50 amino acids from the N-terminus and by 7 amino acids from the C-terminus. Plasmids (pET200-derivatives) containing cDNA of TROSPA substitution mutants (TROSPA V1, V2 and V3) were produced according to the *Molecular Cloning* mutagenesis protocol [24] using *I. ricinus* TROSPA cDNA in pET200 as a template. The following pairs of primers were used for PCR to produce particular TROSPA mutants: T1F and T1R to obtain TROSPA V1, T2F and T2R to obtain TROSPA V2, T3F and T3R to obtain TROSPA V3. TROSPA V4 and V5 cDNA were ordered from the GeneArt® company and amplified by PCR using TF and TR primers. All the primer sequences are in Materials S1A. The modified TROSPA cDNAs were cloned into the pET expression vector using a Champion™ pET200 Directional TOPO Expression Kit according to the manufacturer’s instructions. As a result, the following pET200 plasmids encoding TROSPA or its mutants were obtained: pET200-TROSPA, pET200-TROSPA_NΔ24, pET200-TROSPA_NΔ44, pET200-TROSPA_NΔ50_CΔ7, pET200-TROSPA_V1, pET200-TROSPA_V2, pET200-TROSPA_V3, pET200-TROSPA_V4, pET200-TROSPA_V5.

### Protein Production and Purification

TROSPA and its mutants were expressed in BL21 Star™ (DE3) One ShotR Chemically Competent *E. coli* according to the manufacturer’s instructions. Briefly, bacterial cells were transformed with the modified pET200 plasmids (encoding wt or mutated TROSPA) by a heat shock method. Then, each transformant was used to inoculate 250 ml of LB medium containing 50 mg/l kanamycin. The cells were grown at 37°C and when the OD_600_ of the culture reached 0.5–0.8, IPTG was added to a final concentration of 0.5 m*M*. After induction, the culture was incubated for 4 h at 37°C. The cell paste was harvested and frozen on dry ice for storage at −20°C. Approximately 10 g of cell paste was resuspended in 20 ml lysis buffer I [50 m*M* Na_2_HPO_4_ pH 8, 50 m*M* NaCl, 1x CelLyticTM (Sigma-Aldrich) MT, 250U Benzonase® Nuclease (Novagen), 1 tablet of Mini EDTA-free Protease Inhibitor Cocktail Tablets (Roche), 0.2 mg/ml lysozyme (BioShop)]. After incubation on ice for 30 min, the lysate was sonicated three times for 30 s on ice. Cell debris was removed by centrifugation and the supernatant was mixed with the same amount of buffer II (50 m*M* Na_2_HPO_4_ pH 7, 300 m*M* NaCl, 30 m*M* imidazole). To purify TROSPA and its mutants, we used affinity chromatography. After protein binding with Ni-NTA resin, the column was washed with 30 m*M* imidazole in buffer III (50 m*M* Na_2_HPO_4_ pH 6, 300 m*M* NaCl, 5%(*v*/*v*) glycerol). Next, the protein was eluted from the column using 200 m*M* imidazole in buffer III. The eluted protein was dialyzed using PBS buffer. To remove the N-terminal polyhistidine-tag, a Enterokinase Cleavage Capture Kit (Millipore) was used according to the manufacturer’s instructions. After digestion, the sample was once again applied to a Ni-NTA charged column to remove the tag and any undigested protein. The first flow-through was collected and dialyzed in PBS buffer. The sequence of TROSPA was confirmed by MALDI-TOF MS. Finally, rabbit TROSPA-antiserum was produced by Eurogentec (according to an 87 day standard protocol) using the highly purified TROSPA protein provided by us.

### Analysis of TROSPA-OspA Interaction

ELISA microplates were coated with TROSPA or TROSPA mutants solution (c = 5 µg/ml) in PBST buffer (PBS buffer containing 0,05% Tween-80) and incubated overnight at 4°C. The microplates were washed five times with PBST using ImmunoWash (Model 1575, Bio-Rad), and the nonspecific binding sites were blocked with 3% BSA in PBST overnight at 4°C. After another five washes with PBST, 100 µl of OspA solutions (serially diluted in PBST, eleven OspA concentrations ranging from 250 µg/ml to 0,2 µg/ml) were applied to each well except for the first vertical row that was composed of blank samples. In a control assay, OspC solution was used instead of OspA. The microplates were incubated for 90 min at room temperature and then washed five times with PBST. Next, 100 µl of anti*-Borrelia* IgG (Abcam) diluted 1∶10000 in PBST containing 1% BSA (PBSTB) was applied to each well. The microplates were incubated for 90 min at room temperature and then washed five times with PBST. Next, 100 µl/well of AP-conjugated anti-rabbit IgG (Abcam) diluted 1∶50000 in PBSTB was applied, and the microplates were incubated for 90 min at room temperature. Finally, the microplates were washed five times with PBST, and the color was developed at room temperature by adding 100 µl/well of AP Substrate Kit (Bio-Rad). The reactions were terminated after 30 min by the addition of 0.4 M NaOH (100 µl/well). Absorbance values were read at 405 nm in a Microplate Reader (Model 550, Bio-Rad). For each TROSPA (wild type or mutant) - OspA (either from *B. garinii, B. afzelii* or *B. burgdorferi s. s*) pair the level of protein binding was determined for eleven OspA concentrations and each individual reaction was repeated 8 times. All experiments were repeated twice. Thus, for TROSPA and each TROSPA mutant the level of OspA binding was determined based on 16 reactions performed for every OspA concentration (2 biological and 8 technical replicates). For each protein pair arithmetic mean of absorbance and SD was calculated for each OspA concentration. Next, the relative level of TROSPA (TROSPA mutant) - OspA binding was calculated. To this end, the arithmetic means of absorbance obtained for all protein pairs at the same OspA concentration were divided by the arithmetic mean of absorbance calculated for OspA from *B. garinii* at corresponding concentration. The relative level of protein binding was expressed in percentage, accordingly, the level of TROSPA binding with OspA from *B. garinii* was considered as 100%.

### Influence of TROSPA-antiserum on TROSPA-OspA Interaction

TROSPA-coated ELISA microplates (prepared as described above) were incubated for 90 min at room temperature with 100 µl/well serially diluted (in PBST) rabbit TROSPA antiserum or rabbit preimmune serum (Eurogentec). Both TROSPA antiserum and preimmune serum were diluted 100 to 51,200 times. One dilution per one microplate column including eight wells was applied. The microplates were washed five times with PBST using Biorad Immunowash at room temperature. Then, 100 µl/well of *B. garinii* OspA solution (c = 30 µg/ml) in PBST was applied and the microplates were incubated for 90 min at room temperature. The microplates were washed five times with PBST, and then 100 µl/well of FITC-conjugated anti*-Borrelia* IgG (Abcam) diluted 1∶500 in PBST was applied. The microplates were incubated for 90 min at room temperature and washed five times with PBST. Fluorescence was measured using a PerkinElmer VICTOR X4 2030 Multilabel Reader. The level of TROSPA-OspA binding was determined for ten TROSPA antiserum/preimmune serum dilutions and each individual reaction was repeated 8 times. All experiments were repeated twice. Thus, to determine the level of OspA binding the arithmetic mean of fluorescence intensity was calculated based on 16 reactions performed for every TROSPA antiserum/preimmune serum dilutions (2 biological and 8 technical replicates).

### Immunization

Wistar rats provided by Department of Toxicology from the Poznan University of Medical Sciences were used for the oral immunization experiments. Groups of five rats were orally immunized three times (on days 0, 14, and 28). Briefly, 200 µg of the purified proteins (either TROSPA or TROSPA, OspA and OspC together) in PBS buffer with or without 1 unit of GEM particles (Gram-positive enhancer matrix, an adjuvant derived from *Lactococcus lactis*, [25]) were added to sodium bicarbonate solution (3.2% w/v) and administered to each rat. Control rats were administered PBS buffer with 1 unit of GEM particles only. The oral administration was performed using a stainless steel feeding needle. After 42 days, blood sampling was performed via cardiac puncture and sera were obtained by centrifugation and stored at −80°C until further analysis.

### IgG Levels in Orally Immunized Rat Sera

The serial dilutions of rat serum in PBST were applied to TROSPA-, OspA- (*B. garinii*) or OspC- (*B. garinii*) coated ELISA microplates (prepared as described above) and incubated for 3 h at room temperature. The microplates were washed five times with PBST followed by application of 100 µl/well of AP-conjugated anti-rat IgG (Invitrogen) diluted 1∶5000 in PBST. The microplates were incubated for 90 min at room temperature. Finally, plates were washed five times with PBST, and the color was developed at room temperature by adding 100 µl/well of BIORAD AP Substrate Kit. The reaction was terminated after 30 min by 0.4 M NaOH addition (100 µl/well). Absorbance was read at 405 nm in a Microplate Reader (Model 550, Bio-Rad). In order to determine the level of IgG the arithmetic mean of absorbance measured in ELISA tests for each serum dilution was calculated. The sera were collected from the group of immunized animals (5 immunized without adjuvant and 5 immunized with adjuvant) from a control group (5 animals immunized only with adjuvant). The levels of IgG were determined for eight serum dilutions and each individual reaction was repeated three times. Thus, for every serum dilution the level of IgG was determined based on 15 reactions (5 biological and 3 technical replicates).

### Statistical Analyses

The two-tailed p-value was determined using an unpaired t-test, and p<0,05 was considered significant.

## Supporting Information

Figure S1 **Comparison of all putative** ***I. ricinus*** **TROSPA sequences from GB.**
(DOC)

Materials S1 **A** - primer sequences. **B** - DNA sequences encoding OspA protein from *B. burgdorferi s. s., B. garinii*, and *B. afzelii*.
(DOC)
