# Correction: Functional Insights into Recombinant TROSPA Protein from *Ixodes ricinus*

**DOI:** 10.1371/annotation/5c0143e4-65c5-4feb-90a6-2824125f9cad

**Published:** 2013-12-03

**Authors:** Marek Figlerowicz, Anna Urbanowicz, Dominik Lewandowski, Jadwiga Jodynis-Liebert, Czeslaw Sadowski

The order of authors is incorrect. Please see the correct author order here: Anna Urbanowicz, Dominik Lewandowski, Jadwiga Jodynis-Liebert, Czeslaw Sadowski, Marek Figlerowicz. 

