# Supplementary material for: Functional Insights into Recombinant TROSPA Protein from Ixodes ricinus
Source: PLoS One. 2013 Oct 18;8(10):e76848. doi: 10.1371/journal.pone.0076848 (PMC3800121; doi:10.1371/journal.pone.0076848)
Supplement: Figure S1 — Comparison of all putative I. ricinus TROSPA sequences from GB. (DOC) [file pone.0076848.s001.doc]

**Fig. S1.** Comparison of all putative *I. ricinus* TROSPA sequences from GB (total number of the sequences is 82, compared using ClustalW). The asterisks mark identical amino acids. The number of identical sequences is indicated at the end of each type of sequence.

10 20 30 40 50 60 70 80 90 100

....|....|....|....|....|....|....|....|....|....|....|....|....|....|....|....|....|....|....|....|

**ABY83459.1** **MAAMEAMAVDMEAMAAAMAAAMVATDTVASSAASAMATEATVAMDTASLSLPLQLSPRSLPQSSLSATAATVATDTVVSSADTEVTDTEDSAATVSATAS**

**ADO64496.1** **----------------------------------------------------------------------------------------------VSDTAS**

**ADO64489.1** **----------------------------------------------------------------------------------------------VSATAS**

**ADO64487.1** **----------------------------------------------------------------------------------------------VSATAS**

**Clustal Consensus ** *****

110 120 130 140 150 160

....|....|....|....|....|....|....|....|....|....|....|....|....|

**ABY83459.1** **LSMLPQLSPRSLPQSSLSATATEASVTADMADTATDTKQFISKGNQHFFAASYLCAWADQSAAGS** (GB:2 identical sequences)

**ADO64496.1** **LSMLPQLSPRSLPQSSLSATATEASVTADMADTATDTKQFISKGNQHFFAASYLCAWADQSAAGS** (GB: 77 identical sequences)

**ADO64489.1** **LSMLPQLSPRSLPQSSLSATATEASVTADMADTATDTKQFISKGNQHFFAASYLCAWADQSAAGS** (GB: 2 identical sequences)

**ADO64487.1** **LSMLPQLSPRSLPQSSLSATATEASVTADMADTATDTKQFISMGNQHFFAASYLCAWADQSAAGS** (GB: 1 sequence)

******************************************** ************************
