# Supplementary material for: Functional Insights into Recombinant TROSPA Protein from Ixodes ricinus
Source: PLoS One. 2013 Oct 18;8(10):e76848. doi: 10.1371/journal.pone.0076848 (PMC3800121; doi:10.1371/journal.pone.0076848)
Supplement: Materials S1 — A - primer sequences. B - DNA sequences encoding OspA protein from B. burgdorferi s. s., B. garinii, and B. afzelii. (DOC) [file pone.0076848.s002.doc]

**Materials S1.**

**A**. Primers:

| name | sequence 5’-3’ |
| --- | --- |
| T5’ | ATGAATTCATGGCGGCTATGGAGGC |
| T3’ | ATCCCGGGTCAACTTCCAGCGGCGC |
| TF | CACCATGGCGGCTATGGAGGC |
| AF | CACCATGAAAAAATATTTATTGGG |
| AR | TTATTTTAAAGCGTTTTTAATTTCATCA |
| CF | CACCATGAAAAAGAATACATTAAGTGCG |
| CR | TTAAGGTTTTTTTGGACTTTCTGCC |
| T24F | CACCACGGACACGGTGGCTTC |
| T44F | CACCGACACGGCGTCGCTGT |
| TR | TCAACTTCCAGCGGCG |
| T50F | CACCCTGCCGCTCCAGTTGTC |
| T7R | TCAGGCCCAAGCGCATAAAT |
| T1F | GGAGGTTACGAACACGCAGAATTCGGCGGCT |
| T1R | AGCCGCCGAATTCTGCGTGTTCGTAACCTCC |
| T2F | GCCACGGCTACGCAGGCTTCGGTTACGGCGAATATGGCGGACAC |
| T2R | GTGTCCGCCATATTCGCCGTAACCGAAGCCTGCGTAGCCGTGGC |
| T3F | GCGGATATGGCGAACACGGCTACGAACACTAAGCAA |
| T3R | TTGCTTAGTGTTCGTAGCCGTGTTCGCCATATCCGC |

**B.** Coding sequences:

*B. burgdorferi sensu stricto* OspA:

ATGAAAAAATATTTATTGGGAATAGGTCTAATATTAGCCTTAATAGCATGTAAGCAAAATGTTAGCAGCCTTGACGAGAAAAACAGCGTTTCAGTAGATTTGCCTGGTGAAATGAAAGTTCTTGTAAGCAAAGAAAAAAACAAAGACGGCAAGTACGATCTAATTGCAACAGTAGACAAGCTTGAGCTTAAAGGAACTTCTGATAAAAACAATGGATCTGGAGTACTTGAAGGCGTAAAAGCTGACAAAAGTAAAGTAAAATTAACAATTTCTGACGATCTAGGTCAAACCACACTTGAAGTTTTCAAAAAAGATGGCAAAACACTAGTATCAAAAAAAGTAACTTCCAAAGACAAGTCATCAACAGAAGAAAAATTCAATGAAAAAGGTGAAGTATCTGAAAAAATAATAACAAGAGCAGACGGAACCAGACTTGAATACACAGAAATTAAAAGCGATGGATCTGGAAAAGCTAAAAAGGTTTTAAAAAGCTATGTTCTTGAAGGAACTTTAACTGCTGAAAAAACAACATTGGCGGTTAAAGAAGGAACTGTTACTTTAAGCAAAAATATTTCAAAATCTGGGGAAGTTTCAGTTGAACTTAATGACACTGACAGTAGTGCTGCTACTAAAAAAACTGCAGCTTGGAATTCAGGCACTTCAACTTTAACAATTACTGTAAACAGTAAAAAAACTAAAGACCTTGTGTTTACAAAAGAAAACACAATTACAGTACAACAATACGACTCAAATGGCACCAAATTAGAGGGGTCAGCAGTTGAAATTACAAAACTTGATGAACTTAAAAACGCTTTAAAATAA

*B. garini* OspA:

ATGAAAAAATATTTATTGGGAATAGGTCTAATATTAGCCTTAATAGCATGTAAGCAAAATGTTAGCAGCCTTGATGAAAAAAATAGCGTTTCAGTAGATTTACCTGGTGGAATGAAAGTTCTTGTAAGTAAAGAAAAAGACAAAGATGGTAAATACAGTCTAATGGCAACAGTAGAAAAGCTTGAGCTTAAAGGAACTTCTGATAAAAACAACGGTTCTGGAACACTTGAAGGTGAAAAAACTGACAAAAGTAAAGTAAAATTAACAATTGCTGAGGATCTAAGTAAAACCACATTTGAAATCTTCAAAGAAGATGGCAAAACATTAGTATCAAAAAAAGTAACCCTTAAAGACAAGTCATCAACAGAAGAAAAATTCAACGAAAAGGGTGAAATATCTGAAAAAACAATAGTAAGAGCAAATGGAACCAGACTTGAATACACAGACATAAAAAGCGATAAAACCGGAAAAGCTAAAGAAGTTTTAAAAGACTTTACTCTTGAAGGAACTCTAGCTGCTGACGGCAAAACAACATTGAAAGTTACAGAAGGCACTGTTACTTTAAGCAAGAACATTTCAAAATCCGGAGAAATAACAGTTGCACTTGATGACACTGACTCTAGCGGCAATAAAAAATCCGGAACATGGGATTCAGGTACTTCTACTTTAACAATTAGTAAAAACAGACAAAAAACTAAACAACTTGTATTCACAAAAGAAGACACAATAACAGTACAAAACTACGACTCAGCAGGCACCAATCTAGAAGGCAAAGCAGTCGAAATTACAACACTTAAAGAACTTAAAGACGCTTTAAAATAA

*B. afzeli* OspA:

ATGAAAAAATATTTATTGGGAATAGGTCTAATATTAGCCTTAATAGCATGCAAGCAAAATGTTAGCAGCCTTGATGAAAAAAACAGCGCTTCAGTAGATTTGCCTGGTGAGATGAAAGTTCTTGTAAGTAAAGAAAAAGACAAAGACGGTAAGTACAGTCTAAAGGCAACAGTAGACAAGATTGAGCTAAAAGGAACTTCTGATAAAGACAATGGTTCTGGAGTGCTTGAAGGTACAAAAGATGACAAAAGTAAAGCAAAATTAACAATTGCTGACGATCTAAGTAAAACCACATTCGAACTTTTCAAAGAAGATGGCAAAACATTAGTGTCAAGAAAAGTAAGTTCTAAAGACAAAACATCAACAGATGAAATGTTCAATGAAAAAGGTGAATTGTCTGCAAAAACCATGACAAGAGAAAATGGAACCAAACTTGAATATACAGAAATGAAAAGCGATGGAACCGGAAAAGCTAAAGAAGTTTTAAAAAACTTTACTCTTGAAGGAAAAGTAGCTAATGATAAAGTAACATTGGAAGTAAAAGAAGGAACCGTTACTTTAAGTAAGGAAATTGCAAAATCTGGAGAAGTAACAGTTGCTCTTAATGACACTAACACTACTCAGGCTACTAAAAAAACTGGCGCATGGGATTCAAAAACTTCTACTTTAACAATTAGTGTTAACAGCAAAAAAACTACACAACTTGTGTTTACTAAACAAGACACAATAACAGTACAAAAATACGACTCCGCAGGTACCAATTTAGAAGGCACAGCAGTCGAAATTAAAACACTTGATGAACTTAAAAACGCTTTAAAATAA
